# The Effects of Calorie Restriction and Exercise on Age-Related Alterations in Corpus Cavernosum

**DOI:** 10.3389/fphys.2020.00045

**Published:** 2020-02-18

**Authors:** Caglar Macit, Unsal V. Ustundag, Ozge C. Dagdeviren, Guldem Mercanoglu, Goksel Sener

**Affiliations:** ^1^Department of Pharmacology, School of Pharmacy, Istanbul Medipol University, Istanbul, Turkey; ^2^Department of Biochemistry, Faculty of Medicine, Istanbul Medipol University, Istanbul, Turkey; ^3^Faculty of Medicine, Department of Biochemistry, Adnan Menderes University, Aydın, Turkey; ^4^Faculty of Pharmacy, Department of Pharmacology, University of Health Sciences, Istanbul, Turkey; ^5^Faculty of Pharmacy, Department of Pharmacology, Marmara University, Istanbul, Turkey

**Keywords:** erectil dysfunction, calorie restriction, physical exercise, aging, nitric oxide

## Abstract

**Background:**

Aging is an important risk factor for erectile dysfunction (ED). Both calorie restriction (CR) and physical exercise (PE) have been established as a non-medical method for the improvement of detrimental changes in aging. It is well documented that both CR and PE influence on sympathetic and parasympathetic systems; however, there are few studies on non-adrenergic non-cholinergic pathways. This study aims to investigate the NO-mediated mechanisms of CR and PE on corpus cavernosum in aged rats.

**Materials and Methods:**

3 and 15 month-old rats were divided into five experimental groups: young rats fed ad libitum (Y-C), aged rats fed ad libitum (O-S), aged rats with CR (O-CR), aged rats with PE (O-PE), and aged rats with CR and PE (O-CR-PE). CR was applied to animals as a 40% reduction of daily food intake for 6 weeks. PE was moderate swimming at 30 min at 3 days/week. The effects of CR and PE were evaluated by histologic, biologic, and in-vitro tissue bath studies.

**Results:**

The outcomes in CR and PE groups (characterized by decreased nitrosative damage together with increased antioxidant capacity) were improved in comparison to the O-S. Apoptotic biomarkers were also lower and both endothelial and smooth muscle cell’ functions were preserved too. There was no statistical difference between apoptosis, antioxidant capacity, and nitrosative damage parameters. Contractile responses to phenylephrine and relaxation responses to carbachol were: O-CR > O-PE > O-CR-PE. In these groups, NOS protein levels determined by western-blot were: eNOS: O-CR = O-CR + PE > O-PE; iNOS: O-CR = O-PE > O-CR-PE; nNOS: O-PE > O-CR-PE > O-CR.

**Conclusion:**

In our study, both CR and PE prevented age-related changes in the corpus cavernosum of rats. Reducing nitrosative damage in the neurovascular structure was the main mechanism. CR and exercise restored the endothelial and smooth muscle cells in corpus cavernosum by decreasing apoptosis. The mechanism of enhancing functional response in corpus cavernosum with CR was the improvement of endothelial function via eNOS activation however it involves increases in the NO-cGMP signaling pathway by an endothelium-independent mechanism with PE. This might be a direct stimulation of smooth muscle cells by NO, which released from the cavernous nerve endings via nNOS activation.

## Introduction

Erectile dysfunction (ED) is one of the most common disorders in males (Kaya et al., 2017). The prevalence of ED in the general population ranges from 30 to 65% in men aged 40–80 years (Campbell et al., 2019). The Massachusetts Male Aging study emphasized the close association between aging and ED by showing an almost 1.5 times increased prevalence of ED after the age of 70 years (Feldman et al., 1994).

The development of a penile erection can simply be summarized in two sequential steps: (1) the transportation of blood into the cavernosal sinusoids resulting in the enlargement and rigidity of the penis, and (2) the reduction of venous outflow via veno-occlusion to maintain the enlargement and rigidity of the penis (Ferrini et al., 2017). However, this two-step process depends on a complex balance and coordination of neurogenic, vascular, and humoral events (Jang et al., 2017), such as the parasympathetic, sympathetic, and nitrergic nerves, neurotransmitters, blood vessels, and cavernous muscles (Claudino et al., 2004). Nitric oxide (NO) plays a key role in this coordination, because the increase in the blood flow via dilation of arterial vessels as well as the increase in the size of corporal sinusoids via the relaxation of smooth muscles is regulated by the NO (Ferrini et al., 2017). Both the nitrergic fibers and vascular endothelial cells supply the corpus cavernosum with NO (Claudino et al., 2004). Decreased nerve and endothelium-mediated corpus cavernosum relaxation, reductions in smooth muscle cell content, and pathological remodeling of the pudendal artery were shown in age-related ED. Moreover, they were attributed largely to increased oxidative stress and endothelial dysfunction (Johnson et al., 2011). However, the underlying molecular mechanisms have not been fully identified (Johnson et al., 2011). Furthermore, conflicting results have been reported, especially in relation to NO-mediated mechanisms (Ferrini et al., 2001; Gonzalez and Rajfer, 2004; Johnson et al., 2011).

A causal connection between oxidative-stress, aging and age-related pathologies (Davalli et al., 2016), as well as the beneficial effects of calorie restriction (CR) and regular physical exercise (PE) on oxidative stress in age-related pathologies is known (Testa et al., 2014; Simioni et al., 2018). Again, the beneficial effects of CR and PE on erectile function have also been shown separately (Ferrini et al., 2001; Claudino et al., 2004; Gonzalez and Rajfer, 2004; Johnson et al., 2011; Testa et al., 2014; Davalli et al., 2016; Souza et al., 2017; Simioni et al., 2018). This study aims to investigate the NO-mediated mechanisms of CR and PE on the corpus cavernosum in aged rats.

## Materials and Methods

### Animals and Study Protocol

A sample of 3-month-old young and 15-month-old aged male Wistar albino rats were divided into five groups of 15 each: young rats fed *ad libitum* (Y-C); aged rats fed *ad libitum* (O-S); aged rats with CR (O-CR); aged rats that were exercise trained (O-PE); and aged rats with CR and that were exercise trained (O-CR-PE).

The *ad libitum* groups of animals were fed with standard chow pellets (composed of 21% soybean protein, 15% sucrose, 43.7% dextrin, 10% corn oil, 0.15% a-methionine, 0.2% choline chloride, 5% salt mix, 2% vitamin mix, and 3% Solka-Floc fiber) and had free access to food and water. CR was applied to animals as a 40% reduction of daily food intake for 6 weeks (Whidden et al., 2011). Exercise training was moderate swimming for 30 min 3 days/week for 6 weeks (Leite et al., 2013). At the end of the study period, animals were then decapitated and corpus cavernosum tissues were excised. In half of the rats in each group, the corpus cavernosum was immediately prepared for the *in vitro* contractility studies. In the other half of the rats in each group, samples were stored at −70°C for the measurement of biochemical parameters. Extra tissue samples were fixed in 10% buffered formalin solution and prepared for routine paraffin embedding for histological analysis.

Animals were housed individually in polycarbonate cages with wood chip bedding and maintained in a temperature-controlled room (50–60% of humidity at 24°C) with a 12-h light-dark cycle (lights on at AM 7:00 and off at PM 7:00).

All experimental procedures were done in accordance with the Guide for the Care and the Use of Laboratory Animals published by the US National Institutes of Health. The local animal ethic committee approval was obtained for all experimental procedures.

### Tissue Antioxidant Capacity and Oxidant Status

While superoxide dismutase (SOD) and glutathione (GSH) levels were measured for antioxidant activity; malondialdehyde (MDA) as a marker of lipid oxidation and 8-hydroxyguenosine (8-OHdG) as an indicator of oxidative DNA damage were measured in tissue samples homogenized in ice-cold 10% trichloroacetic acid (TCA). MDA was determined spectropohometrically by measuring the presence of thiobarbituric acid-reactive substances (Okhawa et al., 1979). SOD enzyme activity determination was done based on the production of H_2_O_2_, from xanthine by xanthine oxidase, and the reduction of nitro blue tetrazolium as previously described (Aladag et al., 2003). 8-OHdG was measured by ELISA (OxiSelect Oxidative DNA Damage Elisa Kit, Cell Biolabs) according to the manufacturer’s instruction. The DNA isolation from the samples was performed by a commercial kit, according to the manufacturer’s instruction (PureLink^®^ Genomic DNA Mini Kit, Life Technology).

### Tissue Nitric Oxide (NO), Peroxynitrite (ONO_2_^–^), and Cyclic Guanylate Cyclase (cGMP) Levels

Nitric oxide was measured in the tissue supernatants as nitrite/nitrate (NOx) concentration by spectrophotometry (Roche, United States). Briefly, tissues were harvested, washed in cold 5% TCA, suspended with an ice-cold assay buffer (ready to use), homogenized on ice, and centrifuged for 2–5 min at 4°C. The supernatant was collected in a clean tube, deproteinized, and neutralized with ice cold 4 M PCA and 2 M KOH, respectively. Samples were added to wells, nitrate reductase was added to convert nitrate to nitrite, and colored with Griess Reagent. The intensity of the color was measured against the standard at 550 nm by spectrophotometer.

To assess the activation of either NO/ONO_2_^–^ or NO/cGMP pathway, ONO_2_^–^ and cGMP levels were measured. For the cGMP measurement, frozen samples were homogenized with 5% TCA and centrifuged. The Supernatant was extracted with water-saturated ether and dried. Reconstituted samples were measured by using an enzyme-linked immunoassay (ELISA) using a commercial kit (ADI-900-013, Enzo Life). ONO_2_^–^ measurement was performed by enzyme-linked immunoassay (ELISA) using a commercial kit (HycultBiotech, PA, United States) according to the manufacturer’s instruction. Accordingly, tissue homogenates were incubated with a biotynlated tracer antibody and transferred to a microplate precoated with nitrated-HSA and allowed to incubate. After incubation, the plate was washed to remove excess traces of antibody. Streptavidin peroxidase followed by TBM was added to the plate to facilitate color development. The reaction was stopped using oxalic acid and color intensity was measured against the standard at 450 nm by spectrophotometer. Tissue protein levels were determined using the method proposed by Folin-Lowry (Lowry et al., 1951).

### Apoptotic/Anti-apoptotic Biomarkers

Western blot was used to detect the change of apoptosis-related proteins. Accordingly, the lysis of Adipose-Derived Stem Cells (ADSCs) exploited the Laemmli Sample Buffer (Bio-Rad). After centrifugation at 4°C, protein components were attained and determined by using a BCATM Protein Assay Kit (Thermo Scientific). Proteins were loaded in sodium dodecyl sulfate-polyacrylamide gel electrophoresis. Obtaining discrete proteins were then transferred to nitrocellulose membranes and incubated overnight at 4°C with primary antibodies of Bcl-xL (sc-892, Santa Cruz), Caspase-3 (sc-7148, Santa Cruz), Bax (sc-7480, Bax Antibody (B-9), Santa Cruz), and p53 (sc-6243, Santa Cruz) over 1 h at room temperature with corresponding secondary antibodies. β-actin was used as a reference protein.

### Total Nitric Oxide Synthase (NOS) Activity and NOS Isoforms

Tissue NOS activity was detected by ELISA (ENOS-100, EnzyChrom^TM^ Nitric Oxide Synthase Assay Kit, Bioassay Systems) according to the manufacturer’s instruction.

The NOS isoforms, nNOS (Sc-5302, NOS1 (A-11) Antibody, Santa Cruz), iNOS (Sc-7271, NOS2 Antibody (C-11), and eNOS (Sc-654 NOS3 Antibody (C-20), Santa Cruz), were determined by Western blotting in tissue homogenates using specific antibodies as described above.

### Histologic Examination

A mid-portion of each penile segment was harvested and immediately fixed in 10% paraformaldehyde for 24 h at 4°C. Specimens were cut into 5-μm sections, stained with hematoxylin-eosin, and evaluated under the light microscope (Olympus BX51).

### Corpus Cavernosum Function

Contractile responses in organ bath study were used to measure the function of corpus cavernosum. The penis was removed and the excised corpus cavernosum from the rats was dissected free of the tunica albuginea and cut into 2 × 2 × 15 mm strips. Corporeal strips were bathed in Krebs–Henseleit solution (118,14 mM NaCl, 4,7 mM KCl, 2,5 mM CaCl_2_, 25 mM NaHCO_3_, 1,2 mM MgSO4, 1,2 mM KH_2_PO_4_, and 11,1 mM glucose) The strips were mounted in 10-mL organ baths containing Krebs solution at 37°C continuously bubbled with a mixture of 95% oxygen and 5% carbon dioxide (pH 7.4). The tissues were equilibrated for 45 min under a resting tension of 500 mg. Changes in the isometric force were recorded using a PowerLab 400 Data Acquisition System (Software Chart, version 4.2, AD Instruments, Colorado Springs, CO, United States).

To verify the contractile ability of the preparations, 118 mM KCl solution was added to the organ baths at the end of the equilibration period. The contractile responses of the corporeal strips to 10^–9^–10^–3^ M phenylephrine were obtained cumulatively and expressed as the percentage of the maximal contraction induced by 118 mM KCl. After a 30-min washout period, corporeal tissues were contracted with a submaximal dose (10^–5^ M) of phenylephrine. The relaxation responses of the pre-contracted tissues were evaluated by adding increasing concentrations of carbachol (10^–9^–10^–3^ M) (Paskaloglu et al., 2004). Potency (pEC_50_) presented as a log of molar concentration to produce 50% of the maximal contractile response elicited by an agonist relative to KCl (118 mmol/L)-induced contraction.

### Statistical Analysis

Statistical analyses were performed with SPSS 24.0 software program (Chicago, IL, United States). All variables were expressed as mean ± SEM. Differences between groups were analyzed by ANOVA and *Post hoc* Bonferroni test and *p* < 0.05 was considered to be statistically significant.

## Results

### Tissue Antioxidant Capacity and Oxidant Status

Both tissue SOD and GSH levels were low in the O-S compared to the Y-C (*p* < 0.01). Parallel with these findings tissue MDA and 8-OHdG levels were high in the O-S group too (*p* < 0.01).

Compared O-S, both CR, and PE trained groups were characterized with decreased oxidative damage (characterized by increased SOD and GSH) together with increased antioxidant capacity (characterized by decreased cavernosal MDA and 8-OHdG levels) (*p* < 0.01 for all comparisons). There was no statistically significant difference between the CR, PE, and CR + PE groups (*p* > 0.05 for all comparisons) (Table 1).

**TABLE 1:** Tissue oxidative status and the antioxidant capacity.

Group	Antioxidant capacity		Oxidative status	
	SOD	GSH	MDA	8-OHdG
	(U/gr tissue)	(μmole/gr tissue)	(nmole/gr tissue)	(ng/mg DNA)
Y-C	7.46 ± 0.32	2.48 ± 0.21	8.43 ± 0.24	1.42 ± 0.14
O-S	3.06 ± 0.13^∗^	0.96 ± 0.10^∗^	12.53 ± 1.23^∗^	3.47 ± 0.20^∗^
O-CR	4.08 ± 0.25^*,&^	2.24 ± 0.15^&^	8.71 ± 0.52^&^	1.87 ± 0.17^&^
O-PE	4.38 ± 0.16^*,&^	2.26 ± 0.08^&^	8.23 ± 0.21^&^	1.78 ± 0.27^&^
O-CR-PE	4.36 ± 0.09^*,&^	2.19 ± 0.19^&^	8.32 ± 0.18^&^	1.63 ± 0.30^&^

**p* < 0.01 ^∗^: compared to Y-C; &: compared to O-S; *n* = 6. Y-C: young control; O-S: old sedentary; O-CR: old calorie restricted; O-PE: old exercise treated; O-CR-PE: old calorie restricted and exercise treated.*

### Tissue NO, ONO_2_^–^, and cGMP Levels

Tissue NO, ONO_2_, and cGMP levels are given in Table 2. Compared to Y-C, in the O-S group NO, ONO_2_^–^ levels were increased, while cGMP levels were significantly depressed (*p* < 0.05). On the other hand, both CR and PE treatments reduced the tissue NO and ONO_2_^–^ levels significantly (*p* < 0.05) and prevented the reduction in tissue cGMP levels (*p* < 0.05). However, there was no statistically significant difference in NO, ONO_2_^–^, and cGMP levels between the CR, PE, and CR + PE groups (*p* > 0.05 for all comparisons).

**TABLE 2:** Tissue nitric oxide, peroxynitrite and cyclic guanylate cyclize levels.

Parameter	Y-C	O-S	O-CR	O-PE	O-CR-PE
NOx (nmol/mg protein)	145 ± 7.2	348 ± 22.3^∗^	188 ± 11.6^*,&^	193 ± 20.3^*,&^	175 ± 12.4^*,&^
ONO_2_^–^(nmol/g tissue)	114 ± 3.1	208 ± 18.2^∗^	150 ± 32.3^*,&^	164 ± 29.8^*,&^	134 ± 24.9^*,&^
cGMP (pmol/mg protein)	12.98 ± 0.32	8.57 ± 0.12^∗^	11.41 ± 0.25^&^	11.03 ± 0.12^&^	12.24 ± 0.18^&^

**p* < 0.05 ^∗^ compared to Y-C; ^&^ compared to O-S; *n* = 6. NO, nitrite/nitrate, ONO_2_^–^, peroxynitrite, cGMP, cyclic guanylate cyclize; Y-C, young control; O-S, old sedentary; O-CR, old calorie restricted; O-PE, old exercise treated; O-CR-PE, old calorie restricted and exercise treated.*

### Apoptotic Biomarkers

**FIGURE 1:**
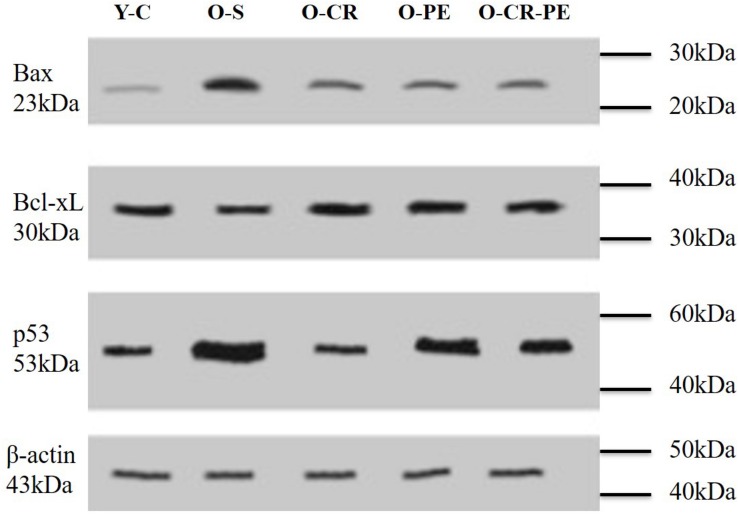
Anti-apoptotic and apoptotic protein bands determined by Western blotting.

**FIGURE 2:**
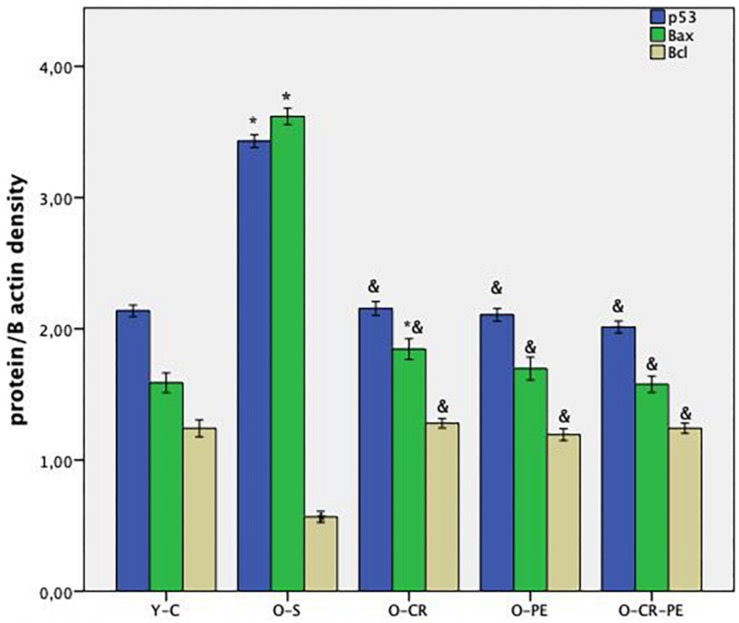
Anti-apoptotic and apoptotic protein levels. *P* < 0.05 ^∗^ compared to Y-C; & compared to O-S; *n* = 6 Y-C, young control; O-S, old sedentary; O-CR, old calorie restricted; O-PE, old exercise treated; O-CR-PE, old calorie restricted and exercise treated.

Apoptosis-related protein bands determined by Western blotting and protein levels that were calculated by comparing the density of the control protein, β-actin, are shown in Figures 1, 2, respectively. As shown in Figure 2, the O-S group was characterized by increased pro-apoptotic (Bax, p53) and decreased in anti-apoptotic protein (Bcl-XL) levels (*p* < 0.001). On the contrary, compared to the O-S group, CR, and PE groups were characterized by increased anti-apoptotic (Bcl-XL) and decreased pro-apoptotic (Bax, p53) protein levels. There were no differences between the PE, CR, and CR + PE groups also (*p* > 0.05 for all comparisons).

### Total NOS Activity, NOS Isoforms, and Histologic Assessment

**FIGURE 3:**
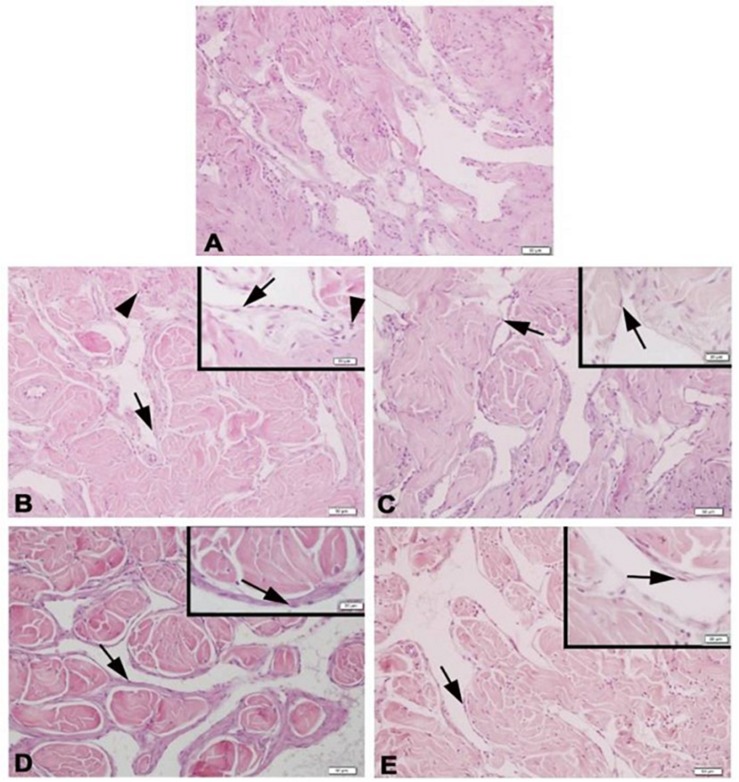
Photomicrographs of corpus cavernosum Normal corpus cavernosum morphology in Y-C group **(A)**; Desquamous endothelial cells and inflammatory cells in O-S group **(B)**; Endothelial and smooth muscle cells of O-CR group **(C)**; Endothelial and smooth muscle cells of O-PE **(D)**; Endothelial and smooth muscle cells of O-CR-PE group **(E)**. arrow: endothelium. arrowhead: inflammatory cells.

Histologic slices of the corpus cavernosum of the Y-C group of animals with normal morphology were characterized by regular endothelial and smooth muscle cells bordered by a layer of dense collagenous (tunica albuginea) and trabecular pattern of veins with irregular vascular channels surrounded by collagen, elastic fiber, and smooth muscle (Figure 3). On the contrary, the O-S group of corpus cavernosum tissue was characterized by moderate endothelial and smooth muscle cell degeneration and inflammatory cell infiltration (Figure 3). CR and PE treated groups of slices were almost in normal morphology of corpus cavernosum (Figure 3).

**FIGURE 4:**
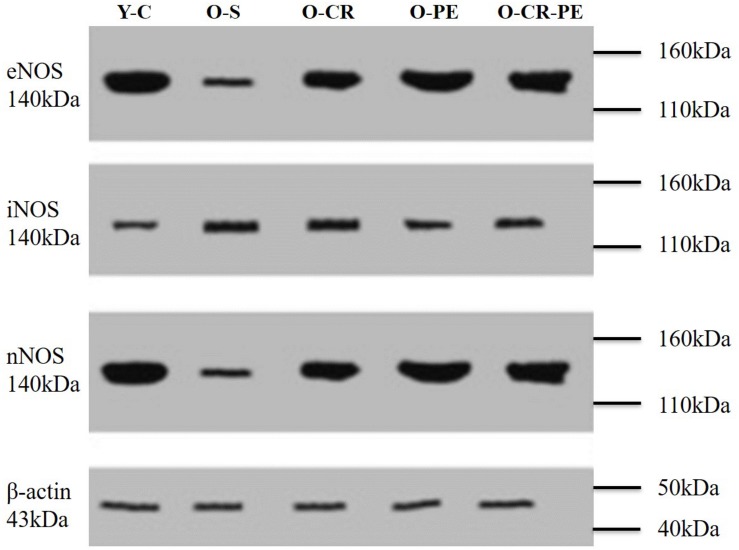
NOS bands determined by Western blotting.

**FIGURE 5:**
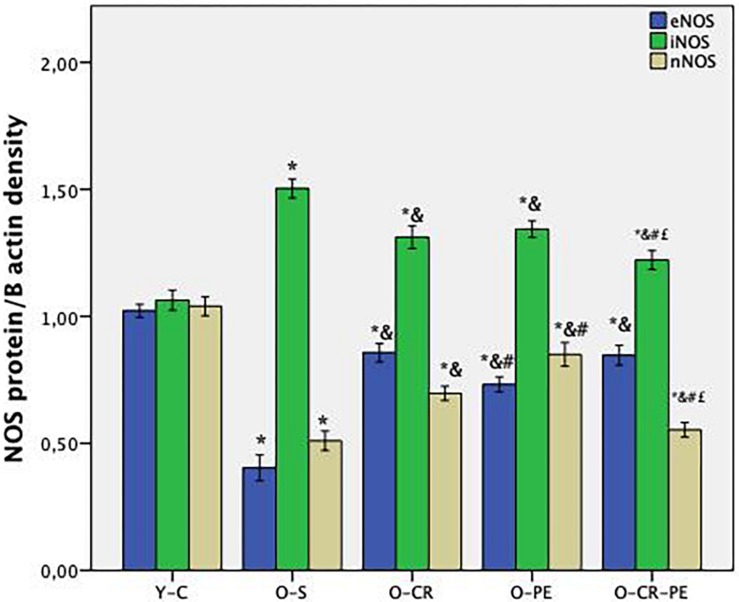
NOS protein levels (*n* = 6) *p* < 0.05 * compared to Y-C; & compared to O-S; # compared to O-CR; £ compared to O-PE; Y-C, young control; O-S, old sedentary O-CR, old calorie restricted O-PE, old exercise trained; O-CR-PE, old calorie restricted and exercise trained.

Compared to the Y-C animals, the O-S group of rats was characterized by increased tissue NOS levels together with decreased eNOS and nNOS and increased iNOS proteins (Figure 4). In the CR and PE trained groups, NOS protein levels determined by Western-blotting were eNOS: O-CR = O-CR-PE > O-PE; iNOS: O-CR = O-PE > O-CR-PE; and nNOS: O-PE > O-CR-PE > O-CR (Figure 5).

### Corpus Cavernosum Function

**FIGURE 6:**
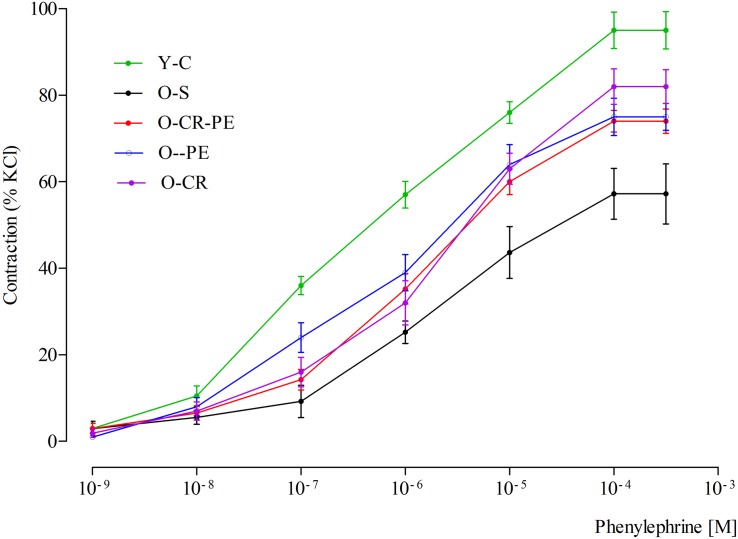
Contractile responses to phenylephrine.

In the pre-contracted (118 mM KCl) corpus cavernosum strips of Y-C, cumulatively added phenylephrine (10^–9^–10^–3^ M) caused a concentration-dependent contraction with a pEC_50_ of 6.26 ± 0.05 (Figure 6). By contrast, in the O-S group, the contractile response of corpus cavernosum to phenylephrine was decreased, causing a significant effect on pEC_50_ (5.65 ± 0.02, *p* < 0.05). Both CR and PE treatment prevented the contractile response of corpus cavernosum to phenylephrine (*p* < 0.05 for all comparisons) (Figure 6). Contractile responses to phenylephrine calculated as pEC_50_ were: O-CR > O-PE > O-CR-PE (Table 3).

**TABLE 3:** Potency obtained from the concentration-response curves.

	pEC_50_				
	Y-C	O-S	O-CR	O-PE	O-CR-PE
Phenylephrine	6.26 ± 0.05	5.65 ± 0.02*	6.04 ± 0.03^&^	5.92 ± 0.04*^,&,^	5.74 ± 0.02*^,&,#,£^
Carbachol	6.49 ± 0.03	6.04 ± 0.04*	6.76 ± 0.04*	6.52 ± 0.05^#^	6.26 ± 0.03*^,#,£^

*p* < 0.05, * compared to Y-C; ^&^ compared to O-S; ^#^ compared to O-CR; ^£^ compared to O-PE; *n* = 6. Potency (pEC_50_) represented as log of molar concentration to produce 50% of maximal contractile response elicited by agonist relative to KCl (118 mmol/L)-induced contraction.

**FIGURE 7:**
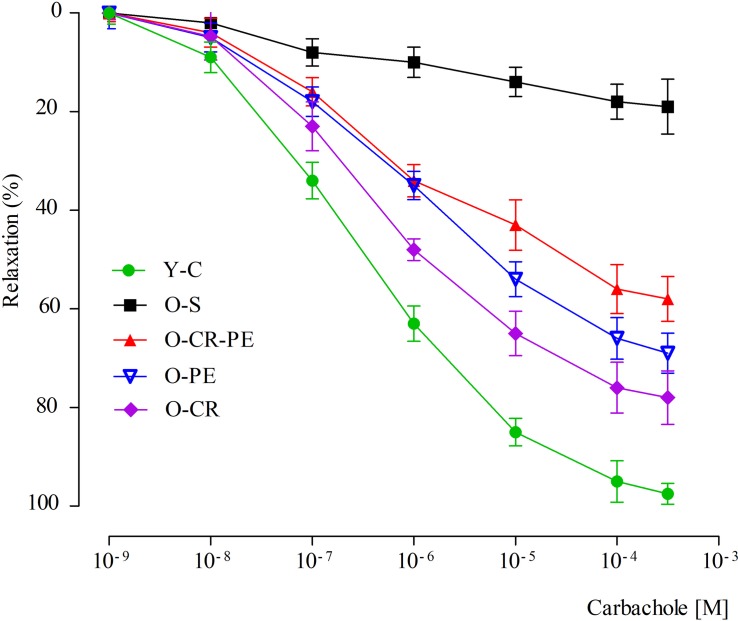
Relaxation responses to carbachol.

Cumulatively added carbachol (10^–9^–10^–3^ M) to corporal tissues, which were pre-contracted with a submaximal dose of phenylephrine (10^–5^ M), caused relaxation in Y-C in a dose-dependent manner (6.49 ± 0.03) (Figure 7). Compared to the Y-C group, relaxation responses to carbachol were markedly reduced (pEC50 = 6.04 ± 0.04, *p* < 0.05) in the O-S group. In the CR and PE treated groups, however, the relaxation responses were higher (Figure 7). Relaxation responses to carbachol calculated as pEC_50_ were O-CR > O-PE > O-CR-PE (Table 3).

## Discussion

It is well known that aging is closely associated with ED with significantly increased prevalence after the age of 70 years (Feldman et al., 1994). Here, we also showed that erectile function in aged rats was significantly impaired compared with their young littermates, as was evident by decreased cavernosal function.

Penile erectile tissue is composed of two corporal bodies called the corpora cavernosa. These cavernosal bodies are composed of sinusoidal spaces with trabecular meshwork, which are lined by endothelium (Agarwal et al., 2006). A penile erection is a two-step mechanical process summarized as: (1) transportation of blood into the expanded cavernosal sinusoids, which results in the enlargement and rigidity of the penis for the initiation of the erection, and (2) maintenance of that enlargement and rigidity by cavernosal veno-occlusion (CVO) in order to prevent the leakage of the blood from the veins before the sexual act is completed (Johnson et al., 2011). In the first step, while the blood flows into the corporal sinusoids via cavernosal arteries, corporal sinusoids expand to provide a space for the pooling of the blood. In the second step, the increase in the intra-corporal pressure via pooling of the blood into the expanded sinusoids shuts the venous channels and decreases the outflow to prevent the leakage of the blood from the vein. The increase in the inflow of the blood via cavernosal arteries, located within the corporal bodies, and expansion of the corporal sinusoids is dependent on the relaxation of smooth muscles located both in the arterial system and corporal sinusoids (Lue and Tanagho, 1987). Therefore, a penile erection is a well-coordinated neuro-vascular process, which includes closed functional interaction between the penis vascular and nervous system (Andersson, 2011). NO plays an important role in this coordination via both nitrergic fibers and vascular endothelial cells (Ferrini et al., 2017; Jang et al., 2017). NO synthesized by nNOS, located in the terminal axons of the nerve innervating the corporal smooth muscle, enters the corporal smooth muscle cell and activates guanylyl cyclase to form cGMP from GTP and induces smooth muscle relaxation and initiates the erection (Burnett et al., 1992). The length of time of erection is dependent on the length of time for maintaining the corporal muscle in its relaxed state. Since the erection requires a balance between inflow and outflow of the blood within the corporal sinusoids, NO produced by eNOS plays a role both in the initiation and continuation of a penile erection (Hannan et al., 2010). NO, synthesized from the endothelial cells by eNOS, diffuses into the smooth muscle cells and causes relaxation with a mechanism similar to nNOS. Also, iNOS may originate from the smooth muscle cell, and NO produced by iNOS acts within the mitochondria. However, iNOS-mediated effects in the corpus cavernosum are quite controversial (Wang et al., 2013; Zhao et al., 2016). Although iNOS is generally considered to be expressed in an inflammatory condition, increasing evidence has demonstrated that iNOS has an important role in the protection of the penile corpus cavernosum either by combating the oxidative stress associated with the ongoing apoptotic process (Gonzalez and Rajfer, 2004; Ferrini et al., 2015) or by inhibiting the breakdown of cGMP (Ferrini et al., 2007). In our study, both three isoforms of NOS (eNOS, nNOS, and nNOS) protein levels in the Y-C group of rats were almost the same. Still, iNOS protein levels were almost doubled in the O-S group. Based on this knowledge, we can speculate that iNOS plays different roles in the penile erection depending on the level of the oxidative status of the penis.

It is known that about 15% loss of functional corporal muscle mass leads the symptomatic ED (Agarwal et al., 2006). The aging-dependent decrease in the amount of the functioning corporal smooth muscle, mainly by apoptosis, was shown (Grünewald and Beal, 1999). Again, the progressive loss of anti-apoptotic genes (Bcl-2 and Bcl-x) in the corpus cavernosum of aged rats was also shown (Yamanaka et al., 2002). In line with these studies, we also showed a decrease in the anti-apoptotic protein (Bcl-XL) levels together with the increase in pro-apoptotic protein (Bax, p53) levels in the O-S group. Again, in our study, decreased contraction and relaxation responses were observed in the functional study of the corpus cavernosum. These findings were consistent with the findings of endothelial and smooth muscle cell degeneration in the histological slices in the O-S group.

Oxidative stress is believed to be the primary trigger for apoptosis (Agarwal et al., 2006). Oxidative stress occurs when the cells are exposed to excessive levels of reactive oxygen spices (ROS) resulting from the imbalance between pro-oxidant and protective mechanisms (Zalba et al., 2000). Under physiological conditions, the tissue antioxidant enzymes (SOD, GSH, and catalase) prevent the continuous formation of ROS. For example, the detoxification of ROS by cavernosal endothelial cells by using these enzymes is known (Dobrina and Patriarca, 1986). Although the upregulation of these enzymes is the main compensatory mechanism, sustained high levels of ROS diminish the activity of these enzymes (Ushiyama et al., 2004; Lagoda et al., 2007). Age-related increase in ROS together with a decrease in antioxidant capacity, which is characterized by reduced levels of SOD and glutathione, have been shown in different study settings (Bivalacqua et al., 2003; Ferrini et al., 2004). Consistent with these findings, in our study, the O-S group of animals was characterized with decreased antioxidant capacity (statistically significant decrease in SOD and GSH levels) and increased oxidant status (statistically increase in MDA and 8-OHdG levels). It should be emphasized that high MDA levels are indicative of lipid changes in the lipid matrix and cell membrane, whereas high 8-OHdG levels indicate DNA damage. Thus, DNA damage and lipid peroxidation might be responsible for the damage to the cavernosal structure in our study. The observation of an increased level of pro-apoptotic protein levels together with the decreased antiapoptotic protein levels in the O-S group supports this argument.

It is well known that NO is a highly reactive free radical that reacts with ROS, especially with the superoxide (O_2_^–^), to form ONO_2_^–^ (Beckman and Koppenol, 1996). According to the body of evidence, ONO^–^_2_ affects the corpus cavernosum in four different ways. Firstly, ONO^–^_2_ causes smooth muscle relaxation, which is less potent than the NO, resulting in ineffective relaxation in the corpus cavernosum (Khan et al., 2001). We showed decreased relaxation and increased ONO_2_^–^ together with decreased NO/cGMP levels in the O-S group of animals. Secondly, ONO_2_^–^ increases the incidence of apoptosis in the endothelium, which leads to denudation of endothelium and a further reduction of available NO (Yamanaka et al., 2002; Fan et al., 2012). In the present study, increased NO and ONO_2_^–^ together with decreased cGMP levels were consistent with the increased iNOS and decreased eNOS protein levels in the O-S group. In this group, the decreased anti-apoptotic protein levels together with the increased pro-apoptotic protein levels also supported these findings. Thirdly, reduced availability of NO by ONO_2_^–^ induced the adhesion of the platelets and leukocytes in the vascular endothelium, which caused vasoconstriction and aggravated ED (Jeremy et al., 2000). Lastly, particularly in the presence of inflammation, produced ONO_2_^–^ from a high level of NO can lead to a cytotoxic effect on cavernosal muscle (Wink et al., 1998). In our study, the observation of inflammatory cells in the histologic slices in the O-S group supported the argument that inflammation results in oxidative stress, and that, in increased oxidative stress conditions, the source of the high level of NO is the iNOS induced by inflammation in age-related ED. As discussed earlier, during the low level of oxidative stress, NO produced by iNOS within the smooth muscle cells combats the oxidative stress associated with the ongoing apoptosis. On the contrary, during the high level of oxidative stress, NO produced by iNOS, induced by inflammation, reacts with the reactive oxygen species to form ONO_2_^–^. This causes endothelial dysfunction, vasoconstriction, a decrease in the availability of NO, and the inhibition of SOD; all these lead to ED.

A causal connection between oxidative stress, aging, and age-related pathologies (Davalli et al., 2016) as well as the beneficial effects CR and PE on oxidative stress in age-related pathologies is known (Testa et al., 2014; Simioni et al., 2018). Again, the beneficial effects of CR and PE on erectile function have also been shown separately (Claudino et al., 2004; Souza et al., 2017). In our study, although the contractile response to phenylephrine (an alpha agonist) was attenuated in O-S, this was prevented both in the O-CR and O-PE groups. Moreover, when the CR and PE groups were compared, the response to phenylephrine was significantly higher in the O-CR than in the O-PE group. This might indicate that CR and PE show their beneficial effects on functional responses of corpus cavernosum through different mechanisms. Increased eNOS levels together with increased response to phenylephrine in the O-CR group support that the effect of CR is mediated by eNOS activation. Other researchers reported similar results (Whidden et al., 2011). On the contrary, lower eNOS protein levels together with mild phenylephrine response in the O-PE group shows the involvement of the NO-cGMP signaling pathway in an endothelium-independent manner. We speculate that this could be a direct stimulation of smooth muscle cells by nNOS, which is released from the cavernous nerve endings. Increased nNOS protein levels in this group supports this hypothesis. Similarly, Claudino et al. (2004) reported an eNOS-independent relaxation response in the corpus cavernosum from trained rats. Again, in the present study, alteration in the relaxation responses of corpus cavernosum to carbachol (a muscarinic agonist) also supports our hypothesis.

## Conclusion

In the present study, CR and PE prevented age-related changes in the corpus cavernosum. Reducing nitrosative damage in the neurovascular structure was the main mechanism. CR and PE restored the endothelial and smooth muscle cells in the corpus cavernosum by decreasing apoptosis. The main mechanism of enhancing functional response in the corpus cavernosum with CR was the maintenance of endothelial function via eNOS activation (supported by increased eNOS levels together with the increased response to phenylephrine an alpha-1 agonist in O-CR group). However, it involves an increase in the NO-cGMP signaling pathway in an endothelium-independent manner with PE. This could be a direct stimulation of smooth muscle cells by nNOS, which is released from the cavernous nerve endings (supported by increased nNOS levels and mild response to carbachol a muscarinic agonist). As a result, lifestyle changes, such as CR and PE, should be considered in combination with pharmacological treatment strategies in the treatment of age-related changes in ED.

## Data Availability Statement

All datasets generated for this study are included in the article/supplementary material.

## Ethics Statement

The animal study was reviewed and approved by the Yeditepe University Experimental Animals Ethics Committee.

## Author Contributions

All authors listed have made a substantial, direct and intellectual contribution to the work, and approved it for publication.

## Conflict of Interest

The authors declare that the research was conducted in the absence of any commercial or financial relationships that could be construed as a potential conflict of interest.
